# Novel homozygous silent mutation of *ITGB3* gene caused Glanzmann thrombasthenia

**DOI:** 10.3389/fped.2022.1062900

**Published:** 2023-01-10

**Authors:** Zhengrong Wang, Yuqing Xu, Yixi Sun, Shuang Wang, Minyue Dong

**Affiliations:** ^1^ School of Clinical Laboratory Science, Guizhou Medical University, Guiyang, China; ^2^ Guizhou Prenatal Diagnosis Center, Affiliated Hospital of Guizhou Medical University, Guiyang, China; ^3^ Women's Hospital, School of Medicine, Zhejiang University, Hangzhou, China; ^4^ Key Laboratory of Reproductive Genetics, Ministry of Education (Zhejiang University), Hangzhou, China

**Keywords:** Glanzmann thrombasthenia, integrin αIIbβ3, ITGB3, silent mutation, whole exome sequencing

## Abstract

Glanzmann thrombasthenia (GT) is a rare inherited disease characterized by mucocutaneous bleeding due to the abnormalities in quantity or quality of platelet membrane GP IIb (CD41) or GP IIIa (CD61). GP IIb and GP IIIa are encoded by the *ITGA2B* and *ITGB3* genes, respectively. Herein, we described a 7-year-old Chinese boy of the consanguineous couple who was diagnosed with GT based on the typical clinical manifestations, absence of blood clot retraction and the reduced expression of CD41 and CD61 in platelets. A homozygous silent variant c.1431C > T (p. G477=) of the *ITGB3* gene was identified by the Whole-exome sequencing and confirmed by Sanger sequencing. The variant was predicted to affect the splicing. RT-PCR and sequencing revealed that the variant caused a deletion of 95 base pairs and frameshift, and subsequently created a premature stop codon in exon 10 of *ITGB3* (p. G477Afs*30). It was indicated that the variant c.1431C > T (p. G477=) of *ITGB3* was the cause for Glanzmann thrombasthenia. Our findings expanded the mutation spectrum and provided the information for the genetic counseling, prenatal diagnosis and preimplantation genetic testing (PGT).

## Introduction

Glanzmann thrombasthenia (GT) is an autosomal recessive disorder characterized by mucocutaneous bleeding symptoms due to platelet defects. Ecchymosis, haematomas, petechiae, nose and gum bleeding and menorrhagia are the most common clinical manifestations (1, 2). According to previous publications, the prevalence of GT is estimated to be approximately 1 in million, with increased rates in high-consanguinity areas (3, 4). The main etiology of the disease is due to the qualitative or quantitative reduction of platelet membrane GP IIb/IIIa (integrin αIIbβ3). αIIbβ3 is a common dimeric complex, which promotes platelet adhesion, platelet aggregation, and is involved in hemostasis (5). GP IIb (CD41) and GP IIIa (CD61) are encoded by the *ITGA2B* and *ITGB3* genes, respectively (6). Mutations in the *ITGB3* or *ITGA2B* genes damage the synthesis of GPIIb/IIIa, inhibit platelet aggregation, and subsequently lead to the development of hereditary platelet incompetence (7, 8).

In the current investigation, we described a 7-year-old Chinese boy with GT caused by a homozygous silent mutation of the *ITGB3* gene. The mutation c.1431C > T (p. G477=) was identified by Whole-exome sequencing (WES) and validated by Sanger sequencing. Moreover, RT-PCR and sequencing demonstrated that the mutation created a premature stop codon and the truncation of the ITGB3.

## Materials and methods

### Subjects

The consanguineous couple came to the Department of Reproductive Genetics, Women's Hospital, School of Medicine, Zhejiang University for genetic counseling because they had two children with generalized mucocutaneous bleeding. The first child of the healthy couple was a female who suffered from generalized mucocutaneous bleeding and died when she was 5 years old. The couple provided no detailed medical documents of the first child. The second child was a healthy female. The third child was a male (proband) at 7 years of age at the time of genetic counseling. He presented with generalized scattered skin, petechiae, and spots on his face after his birth. A blood clot retraction test revealed that the clots did not shrink over 24 h. Furthermore, the expression levels of CD41 and CD61 were significantly reduced as compared with control while the expression of CD42 was comparable with control (Supplementary Figure S1).

The use of medical records of this family was approved by the Institutional Review Board of the Women’s Hospital, School of Medicine, Zhejiang University and the participants provided their written informed consents.

### Whole exome sequencing

Genomic DNA was extracted by a QIAamp DNA blood mini kit (Qiagen, Hilden, Germany) according to the manufacturer's instructions and then was fragmented by Covaris LE220 (Massachusetts, USA) to generate a paired-end library (200–250 bp). All amplified libraries were performed on the BGISEQ-500 platform (BGI, Shenzhen, China), the single-strand DNA was mixed with MGIEasy™ DNA Library Prep Kit V1 (BGI, Shenzhen, China) and then sequenced using 100SR chemistry with BGISEQ-500RS high-throughput sequencing Kit (BGI, Shenzhen, China).

Variants were assessed according to the protocol issued by the American College of Medical Genetics and Genomics (ACMG) (Richards et al., 2015). DECIPHER (http://decipher.sanger.ac.uk), OMIM (http://omim.org/), PubMed (http://www.ncbi.nlm.nih.gov/pubmed), ClinVar (https://www.ncbi.nlm.nih.gov/clinvar/), and HGMD (http://www.hgmd.cf.ac.uk/ac/index.php) databases were used to investigate the clinical relevance of the mutations (9).

### Sanger sequencing

Sanger sequencing was carried out to confirm the variant of *ITGB3* gene. The primers were designed using Oligo Primer Designer (Forward: 5′-GATACTATTCCCGTGCTTG-3′; Reverse: 5′-CACATTGACCACAGAGGC-3′). The DNA was amplified using the following procedure: 95°C for 10 min; 35 cycles at 95°C for 30 s, 60°C for 30 s, 72°C for 30 s; 72°C for 10 min. Sequencing was performed by an ABI 3130 DNA analyzer (10).

### Splicing assay

Total RNA was extracted from the peripheral blood cells of proband and a healthy control using TRIzol (Takara, Japan) and reverse-transcribed using RT Kit (Takara, Japan) following the manufacturer's instructions. RT-PCR was performed using GoldStar Best Master Mix (CWBIO, Beijing) with the primers designed using Oligo Primer Designer as following: Forward:5′-AAGATTGGAGACACGGTGAG-3′ and Reverse: 5′-GCAGTAACGGTTGCAGGTAT-3′. The procedure of the PCR was as follows: 94°C for 10 min followed by 35 cycles at 94°C for 30 s, 60°C for 30 s, 72°C for 30 s, and a final extension step at 72°C for 10 min. Sequencing was performed by an ABI 3130 DNA analyzer.

## Results

### Identification of novel silent mutation of *ITGB3*

**Figure 1:**
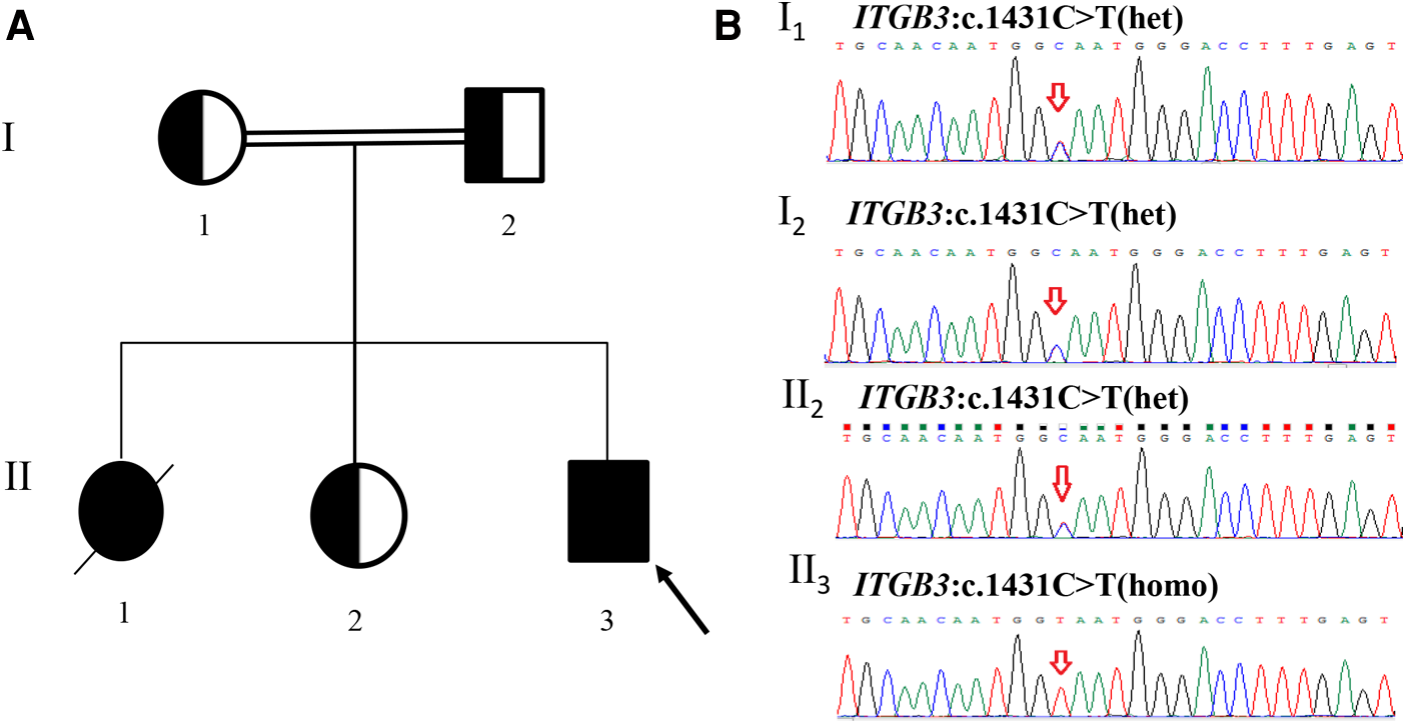
Pedigree of the family and sanger sequencing validation. (**A**) II_3_(proband) had a homozygous mutation (c.1431C > T) in exon 10 of *ITGB3* gene. I_1_(mother), I_2_(father) and II_2_(the second elder sister) were all heterozygous carriers. II_1_ died at 5 years old and did not carry out any genetic test. (**B**) Sanger sequencing results of the family members (red arrows indicate the mutation site).

A homozygous silent mutation on exon 10 of *ITGB3*: c.1431C > T (p. G477=) was identified in proband by WES and confirmed by Sanger sequencing. The parents and the second elder sister were heterozygous carriers, in accordance with the autosomal recessive inheritance pattern (Figure 1). The mutation has never been reported in any database (gnomAD, ClinVar or HGMD) or literature.

### Prediction of the variant c.1431c > T

**Figure 2:**
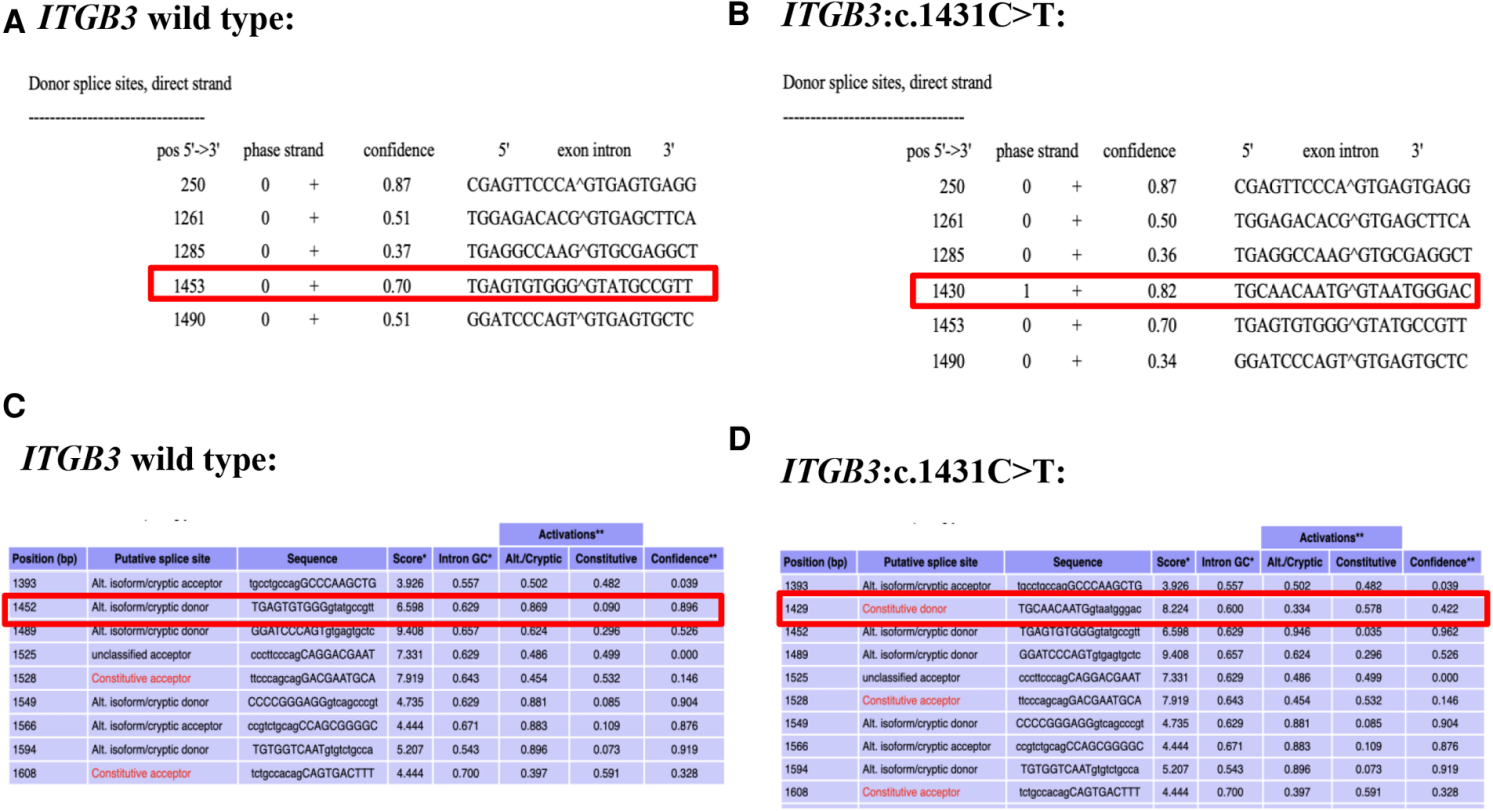
Predict results of the c.1431C > T variant site. (**A**) The predict result of wild type *ITGB3* by using NetGene2 Server (the red square represented). (**B**) The predict result of *ITGB3*: c.1431C > T by using NetGene2 Server (the red square represented). (**C**) The predict result of wild type *ITGB3* by using ASSP (the red square represented). (**D**) The predict result of *ITGB3*: c.1431C > T by using ASSP (the red square represented).

NetGene2 Server (http://www.cbs.dtu.dk/services/NetGene2/) was used to predict the effects of the variation c.1431C > T (p. G477=) on splicing. A new splicing site was found with confidence score of 0.82 (Figures 2A,B). Alternative Splice Site Predictor (ASSP) (http://wangcomputing.com/assp/index.html) was also used to predict the mutation, which was in accordance with the NetGene2 Server (Figures 2C,D).

### Pathogenicity of the variant c.1431c > T

**Figure 3:**
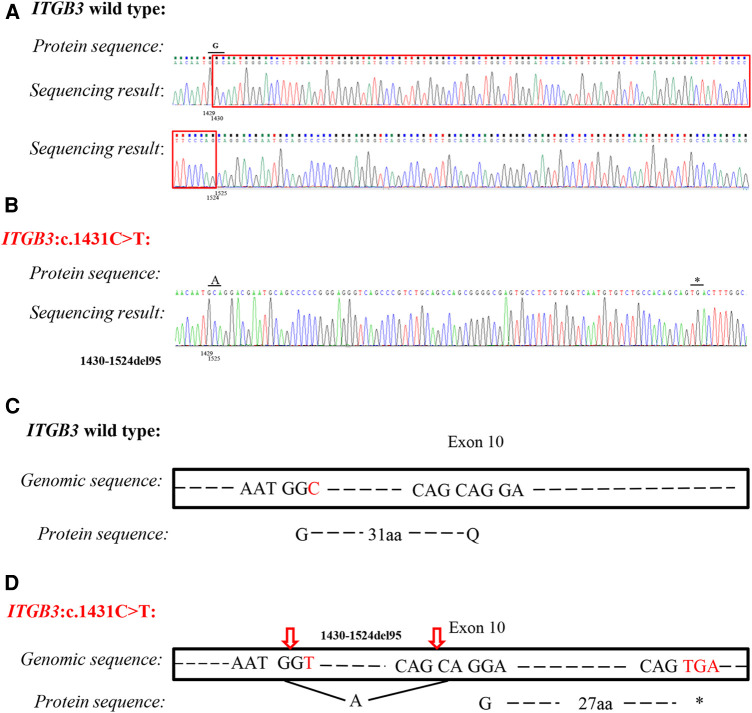
Analysis of silent variant c.1431C > T (p. G477=) of *ITGB3 gene*. Sequencing results of *ITGB3* cDNA from a healthy control (**A**) and proband (**B**). The mutation caused 95 base pairs deletion (c.1430_1524del) and frameshift and then *ITGB3* was truncated by a creation of a premature stop codon. (**C**) Splicing schematic representation of exon 10 organization in wild type of *ITGB3* gene. (**D**) Splicing schematic representation of exon 10 organization in mutant type (*ITGB3*: c.1431C > T). The amino acid encoded at position 477 was changed from Glycine to Alanine, and caused frameshift. *ITGB3* was truncated after 30 amino acids translation (p. G477Afs*30).

Based on the highly consistent genotype–phenotype correlation and splicing prediction, RT-PCR was performed to verify the pathogenicity. With the designed primers, the exon 10 of *ITGB3* was amplified. It was showed that the mutation c.1431C > T resulted in a new donor splice site at the position of 1,430–1,431 and a new acceptor splice site at the position of 1,523–1,524 base pairs, which caused a deletion of 95 base pairs (c.1430_1524del, Figure 3A,B). Therefore, the amino acid encoded at the position 477 was changed from Glycine to Alanine and then frameshift was found. *ITGB3* was truncated by a creation of a premature stop codon after 30 amino acids translation (p. G477Afs*30) (Figure 3C,D). According to ACMG recommendations (11), the mutation *ITGB3*: c.1431C > T (p. G477=) was classified as likely pathogenic.

## Discussion

In the current investigation, we described a Chinese boy with GT due to the homozygous silent mutation of the *ITGB3* gene. A heterozygous mutation was detected in his healthy parents and sister. The silent mutation c.1431C > T (p. G477=) was proved to affect splicing, which resulted in a premature stop codon and truncation of the *ITGB3*. In addition, the CD41 and CD61 of the proband were nearly absent, which significantly damaged the function of αIIbβ3. Therefore, the platelet adhesion, platelet aggregation and clot retraction were affected and consequently caused GT. The mutation has never been reported in any database or literature, indicating our findings expand the spectrum of the diagnosis for the GT and provide insight and information for the genetic counseling.

GT is a rare inherited abnormal platelet function disorder (12, 13). Patients with GT may experience mild to severe bleeding symptoms, including easy bruising, epistaxis, mucosal bleeding, and increased bleeding after trauma or surgery (14, 15). Most of the children may die at their early age due to severe excessive bleeding (3). Heavy menstrual bleeding (HMB) is common in adolescent and adult females (16). In this study, the 7-year-old boy diagnosed with GT presented generalized scattered skin, petechiae and spots on his face after his birth. A blood clot retraction test revealed that the clots did not shrink over 24 h. Furthermore, the expression levels of CD41 and CD61 were significantly lower than those in normal while the expression level of CD42 was the same as healthy people.

GT is caused by the mutations of *ITGA2B* or *ITGB3* genes encoding the αIIbβ3 integrin (6, 11). αIIbβ3 integrin is made up of CD41 and CD61. Low expression of CD41 and CD61 on platelets severely influence platelets functions, including platelet adhesion, platelet aggregation and clot retraction (17, 18). Autosomal recessive inheritance is the general rule. Clinically, people with GT are classified into three groups out of the different expression and function of αIIbβ3. Most patients are originally identified as type I, with less than 5% αIIbβ3. Some people belonging to type II GT express low but residual αIIbβ3 (5%–20%) (19, 20). Furthermore, in type III GT patients, also named variant-type patients, αIIbβ3 expression reaches 100% of normal level while the αIIbβ3 fails to function. The most common subtype is GT type I which accounts for 78% of patients, while GT type II and type III constitute 14% and 8% of cases, respectively (11). As a result of loss of αIIbβ3 function, platelets are unable to bind fibrinogen (Fg) and other adhesive proteins after vessel injury, which may lead to loss of thrombus formation and clot retraction in some cases (21, 22). In the present study, a homozygous silent mutation c.1431C > T (p. G477=) of *ITGB3* gene was identified and confirmed in proband by WES and Sanger sequencing. His parents and sister carried the heterozygous *ITGB3* c.1431C > T (p. G477=) mutation and all of them did not present bleeding symptoms. Based on the genotype–phenotype correlation and the results of the effect on splicing by two online prediction tools (NetGene2 and ASSP), RT-PCR was carried out to identify the pathology of the mutation. It showed that the mutation caused 95 pairs base deletion and a premature termination codon of *ITGB3*, which might lead to absence of ITGB3 protein out of nonsense-mediated decay (NMD) or truncated ITGB3 protein. To our best acknowledgement, the silent mutation c.1431C > T (p. G477=) has never been reported before. Taken together, the homozygous silent mutation c.1431C > T (p. G477=) of *ITGB3* in proband may explain the cause for GT and the proband belongs to the type I GT.

Nowadays, platelet transfusions, anti-fibrinolytic agents infusion (aminocaproic acid or tranexamic), recombinant activated factor VII (rFVIIa) infusion and hematopoietic stem cell transplantation (HSCT) have been the mainstay of therapies for GT patients (23, 24). However, the treatment of GT patients remains unsatisfactory. Patients’ quality of life is significantly impaired by multiple, spontaneous mucocutaneous bleeding episodes, and the high risk of hemorrhaging with surgery or any trauma (3). Because the therapies mentioned above focus on bleeding relief episodes rather than providing a cure except the costly HSCT (3). More importantly, the diagnosis of GT is often overlooked, as it shares common clinical and laboratory features with other platelet disorders. Therefore, it is of great value to carry out genetic diagnosis as early as possible (25). In addition, prenatal diagnosis or PGT is significant in families with GT history.

The gene *ITGB3* was located on chromosome 17q21.32 with 15 exons. According to HGMD (released February 2022), all 223 mutations have been reported in *ITGB3*. Among them, are 144 missense or nonsense mutations (118 pathogenic mutations, 21 uncertain significance mutation and 5 polymorphic mutations), 18 splicing mutations (17 pathogenic mutations and 1 uncertain significance mutation), 3 regulatory substitutions (3 polymorphic mutations), 37 small deletions (36 pathogenic mutations and 1 uncertain significance mutation), 8 small insertions/duplications mutations (pathogenic mutations), 4 small insertions mutation (pathogenic mutations), 4 gross deletions (pathogenic mutations), 4 gross insertions (pathogenic mutations), 1 complex rearrangement (pathogenic mutation). More than 70% of the mutations are associated with GT.

## Conclusion

In conclusion, we report a novel homozygous silent variant c.1431C > T (p. G477=) in exon 10 of the *ITGB3* gene in a GT family by the combined applications of WES, Sanger sequencing and bioinformatics analysis. Furthermore, RT-PCR is necessary to perform if the genotype–phenotype correlation is consistent while only a homozygous silent mutation in autosomal recessive disease is detected. The RT-PCR and sequencing verify that the mutation causes a premature termination codon of the *ITGB3*, upgrading pathogenicity evidence of the silent mutation. These findings are helpful for prenatal diagnosis and preimplantation testing for GT.

## Data Availability

The datasets presented in this study can be found in online repositories. The names of the repository/repositories and accession number(s) can be found in the article/**Supplementary Material**.
