# Supplementary figures and images for: Novel homozygous silent mutation of ITGB3 gene caused Glanzmann thrombasthenia
Source: Front Pediatr. 2023 Jan 10;10:1062900. doi: 10.3389/fped.2022.1062900 (PMC9871544; doi:10.3389/fped.2022.1062900)

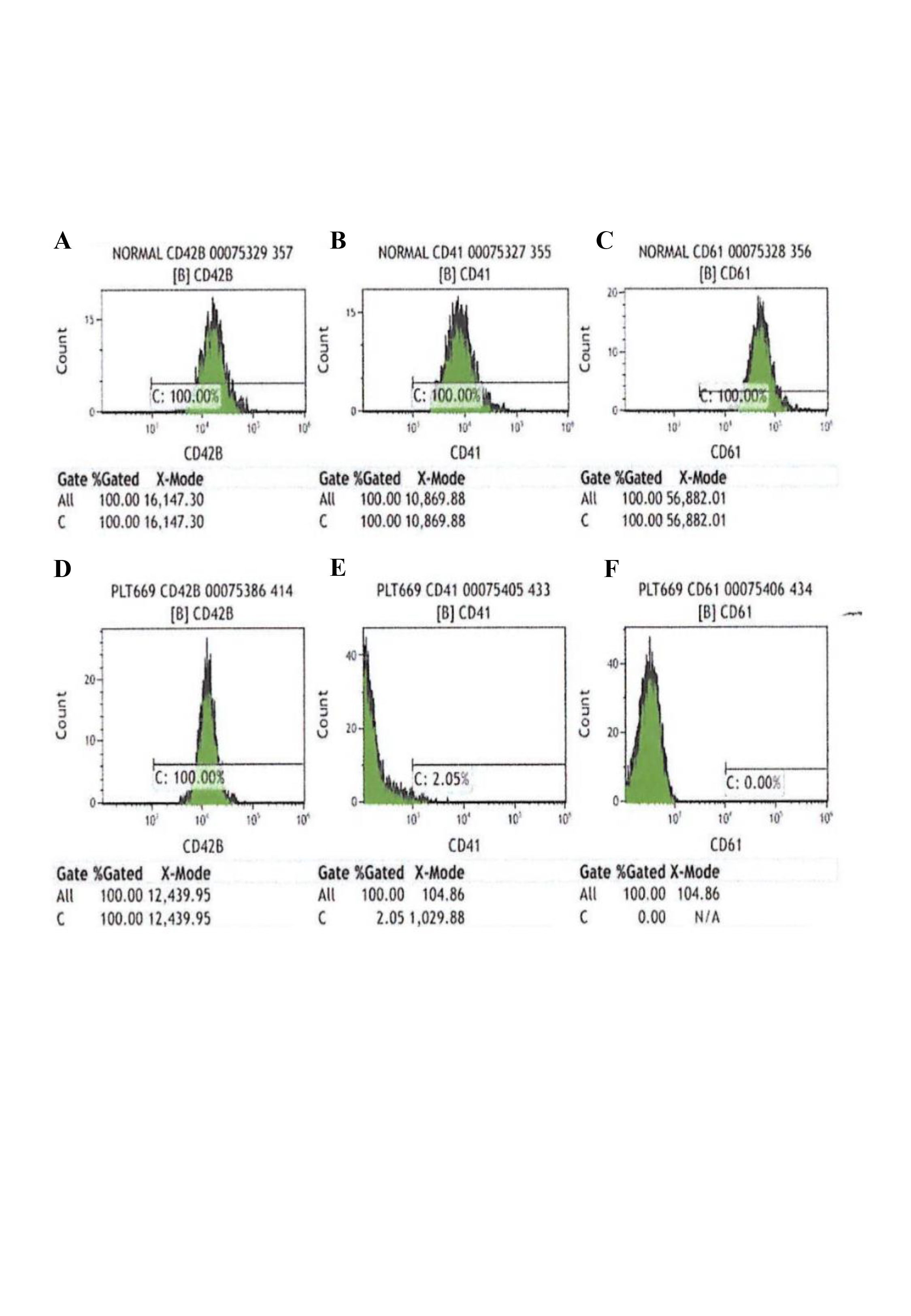

Supplement: Supplementary file 1 [file Image1.tif]
